# Correction to: Circular RNA circ-CSPP1 regulates CCNE2 to facilitate hepatocellular carcinoma cell growth via sponging miR-577

**DOI:** 10.1186/s12935-020-01339-z

**Published:** 2020-07-08

**Authors:** Qian Sun, Rui Yu, Chunfeng Wang, Jianning Yao, Lianfeng Zhang

**Affiliations:** Department of Gastroenterology and Hepatology, The First Affiliated Hospital of Zhengzhou University, 1 Jianshe Dong Lu, Erqi District, Zhengzhou, 450052 Henan China

## Correction to: Cancer Cell Int 20:202 (2020) 10.1186/s12935-020-01287-8

Following publication of the original article [1], the authors notified us that Fig. 5 was incorrect.

The corrected Fig. 5 is presented below.

**Fig. 5:**
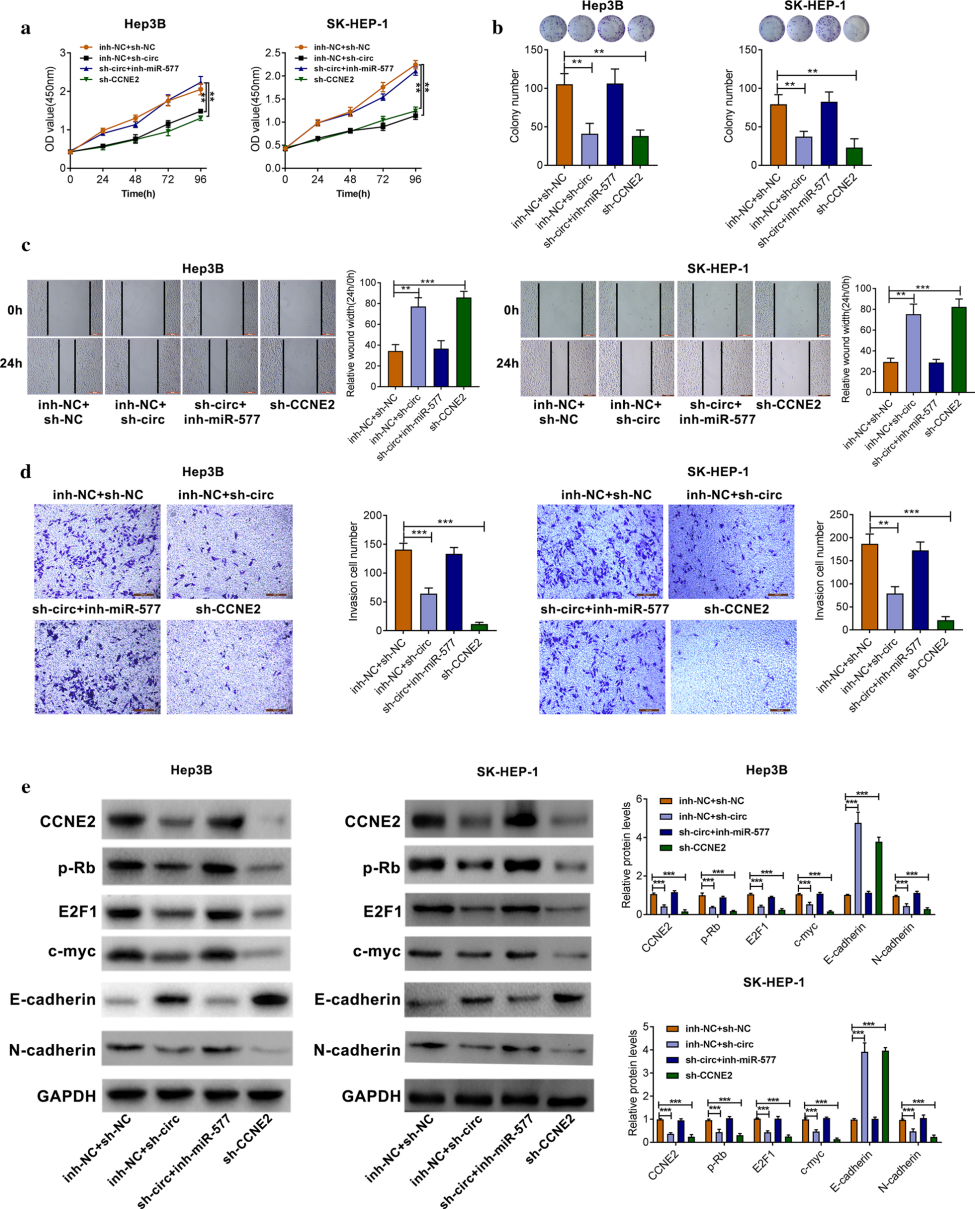
The role of circ-CSPP1 in the progression of HCC was dependent on regulating miR-577/CCNE2. **a** CCK-8 assay was used to determine cell proliferation. **b** The colony formation assay was performed. **c** The migratory ability of cells was decreased by wound healing assay. **d** The transwell assay showed the number of invasive cells. **e** The protein expression levels of CCNE2, p-Rb, E2F1, c-myc, E-cadherin and N-cadherin were detected by Western blotting. *p < 0.05; **p < 0.01 and ***p < 0.001
